# Stability enhancement of clinical grade multipotent mesenchymal stromal cell-based products

**DOI:** 10.1186/s12967-018-1659-4

**Published:** 2018-10-24

**Authors:** Clémentine Mirabel, Eduard Puente-Massaguer, Anna del Mazo-Barbara, Blanca Reyes, Philip Morton, Francesc Gòdia, Joaquim Vives

**Affiliations:** 1 Servei de Teràpia Cellular, Banc de Sang i Teixits, Edifici Dr. Frederic Duran i Jordà, Passeig Taulat, 116, 08005 Barcelona, Spain; 2 Musculoskeletal Tissue Engineering Group, Vall d’Hebron Research Institute (VHIR), Universitat Autònoma de Barcelona, Passeig de la Vall d’Hebron 129-139, 08035 Barcelona, Spain; 3 Albumedix Ltd, 59 Castle Boulevard, Nottingham, NG7 1FD UK; 4 Departament d’Enginyeria Química, Biològica i Ambiental Escola d’Enginyeria, Universitat Autònoma de Barcelona, 08193 Bellaterra, Cerdanyola del Vallès, Spain; 5 Departament de Medicina, Universitat Autònoma de Barcelona, Passeig de la Vall d’Hebron 129-139, 08035 Barcelona, Spain

**Keywords:** Multipotent mesenchymal stromal cell, Potency assay, Cellular therapy, Cell culture, Logistics, Apoptosis, Stability assessment, Quality compliance

## Abstract

**Background:**

Successful delivery of cell-based therapeutics into patients is compromised by their short shelf-life upon release from production facilities due to the living nature of the active component that rapidly loses viability, and therefore its properties. In this context, the use of appropriate additives may contribute to the stabilisation of the cellular component within specifications for a longer time until administration.

**Results:**

In the present study, we evaluated the effect of different formulations on the stability of viability, identity, and potency of clinical grade multipotent mesenchymal stromal cells in suspension, both electrolyte solution and protein content were found to impact on their shelf-life. Particularly cryopreservation of cells in a Plasmalyte 148 supplemented with 2% (w/v) AlbIX (a yeast-derived recombinant albumin) and 10% (v/v) dimethyl sulfoxide, and final formulation post-thawing in Plasmalyte 148 supplemented with 2% (w/v) AlbIX enabling prolonged stability from 24 h up to 72 h in optimal conditions. Further investigation on the mechanisms of action involved revealed a delay of apoptosis progression into late stage when AlbIX was present.

**Conclusions:**

The use of optimal formulations for each cell type of interest is crucial to extend the shelf life of cell-based pharmaceuticals and contribute to solve logistical challenges. We demonstrated that the use of Plasmalyte 148 supplemented with 2% (w/v) AlbIX resulted in superior stability of multipotent mesenchymal stromal cells without affecting their identity and multipotency.

**Electronic supplementary material:**

The online version of this article (10.1186/s12967-018-1659-4) contains supplementary material, which is available to authorized users.

## Background

Over the last decade, research and development on cell-based medicines has undergone a sharp rise bringing with it the necessity of building up a new market of reagents and materials to supply the new clinical applications [1]. However, the use of living cells as an active ingredient poses great challenges, particularly because of their short shelf-life in the final product formulation, which is seriously affected by the rapid loss of viability, within hours from product release [2]. As an alternative to freshly prepared products, cryopreservation allows for the banking of cells thus postponing their expiration. However, freeze/thawing cycles may (A) impact on the safety and efficacy of cells, (B) lead to low cell recovery yields, and (C) post-thawing stability still remains a challenge, since shelf-life of the reconstituted product will fall out of specifications within hours, alike to fresh products. This situation hinders logistics and limits the geographical range for delivery and therefore the widespread use of such therapies. It also requires the conditioning of cells into the final formulation, quality controls, product release and shipping need to be performed very rapidly, in a coordinated manner with patients, physicians and surgery room personnel for immediate administration.

In this context, the biological properties of multipotent mesenchymal stromal cells (MSC) are of great interest to be used in a wide range of clinical applications, such as tissue regeneration or the management of immunological disorders, due to (a) their potential to differentiate into other specialised cell types, (b) their ability to respond to local signalling cues and affect the microenvironment; and (c) their capacity to modulate immune response [3]. Remarkably, ex vivo expansion of homogeneous populations of MSC up to large numbers is feasible within an acceptable time frame and cost, following relatively simple cell culture strategies, therefore making possible to manufacture MSC-based medicinal products in compliance with regulatory and quality requirements for clinical use [4].

The selection of excipients plays a key role in the maintenance of critical quality attributes (CQA) of the final product. This step is even more critical for cell therapy products with human albumin as one of the most popular additives. Human albumin is the most ubiquitous protein in blood and it is present in many tissues and body compartments, acting both as a buffer and as a reservoir for numerous smaller entities such as metals, hormones, fatty acids and toxins [5]. In this sense, albumin shuttles such molecules from tissues of high to low concentration. Additionally, albumin also constitutes about 75% of the colloidal oncotic pressure of blood and the single free cysteine of albumin (at position 34) makes up most of the reducing equivalents present in blood. All these properties are traits which are functional in employing albumin as a long-established ingredient of cell-culture media, facilitating growth of many cell types, but also allowing for high viability rates upon cryopreservation and final formulation of cell-based therapeutics, thus contributing as a tool to overcome challenges in manufacturing, formulation and handling. Historically, albumin has been used in the context of serum, which can be successfully removed from cell culture media and substituted by the addition of albumin. One major handicap of using plasma derived albumin is its limited availability, associated to volunteer donations, with only few countries allowing economical compensation. In addition the variation in worldwide diets affecting plasma pools can lead to variations in albumin batches and inconsistencies when setting up worldwide medical products that use plasma derived albumin. Production of recombinant albumin by means of biotechnological platforms could help circumventing such limitation. In recent years, the positive properties of albumin in cell culture have, in some instances been expanded to its use in cryopreservation, or formulation buffers for stem cell therapies. Recombinant human albumin offers a safe solution for optimized cell performance and as an animal and human component-free product it provides regulatory benefits while improving cell viability and controlling batch-to-batch consistency.

Herein we investigated the impact of main components of the final formulation on MSC-based products, based on International Conference on Harmonisation of Technical Requirements for Registration of Pharmaceuticals for Human Use (ICH) and European Medicine Agency (EMA) recommendations [6, 7], in which the performance of GMP-grade recombinant human albumin and human serum albumin (HSA) as stabiliser of CQA was evaluated using clinical grade Bone Marrow (BM)-derived MSC, aiming at developing longer stability, highly viable, better performing final formulations.

## Results

### Selection of an electrolyte solution

**Fig. 1:**
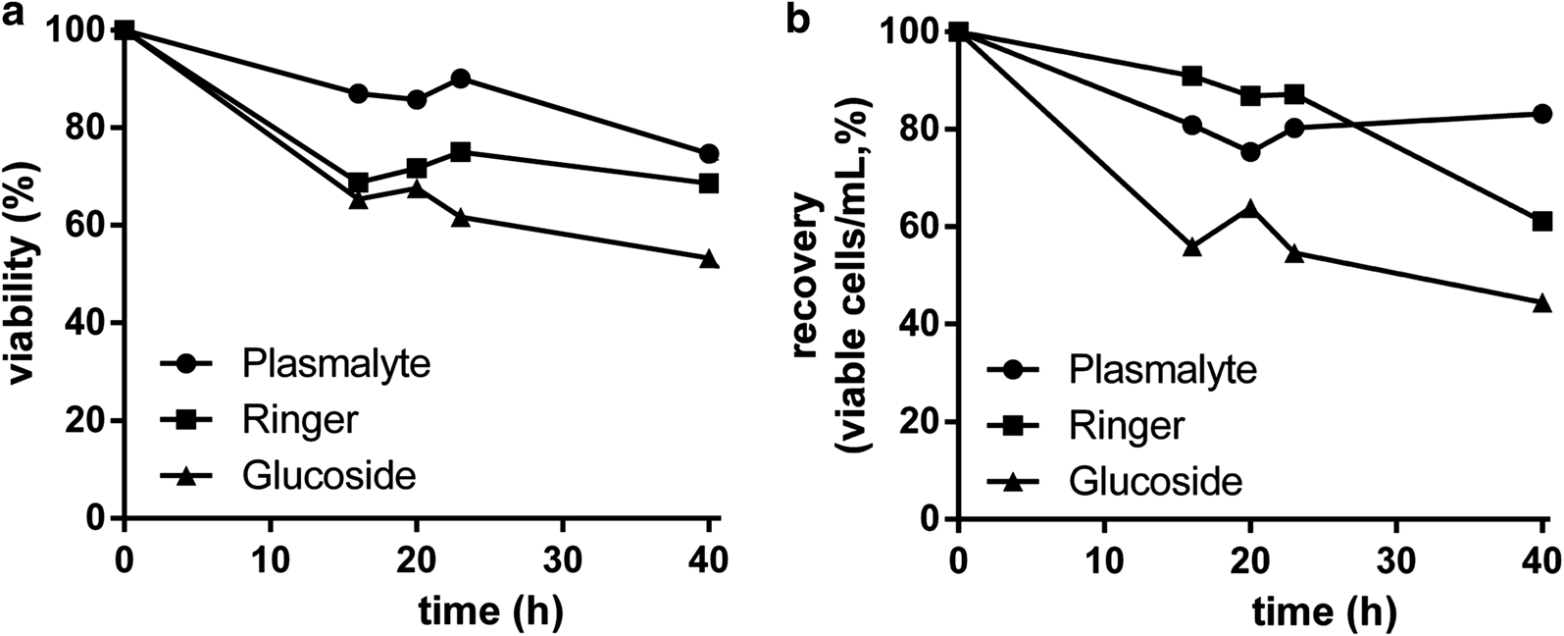
Effect of different electrolyte solutions on the stability of MSC in a syringe. Evolution of cell viability (%) (**a**) and cell recovery (**b**) along time at room temperature using 5% glucoside solution, Ringer lactate solution, and Viaflo plasmalyte 148 supplemented with 2% (w/v) HSA. One sample per condition and time point was tested in duplicates. HSA, human serum albumin; MSC, multipotent mesenchymal stromal cells

In a first set of experiments, ex vivo expanded clinical grade MSC were resuspended in different formulations of isotonic electrolyte solutions (namely, 5% glucoside solution, Ringer lactate solution, and Plasmalyte 148) supplemented with 2% (w/v) HSA in syringes at a final concentration of 7.5 × 10^6^ ± 0.9 × 10^6^ MSC/mL in a final volume of 4.9 ± 0.2 mL (Table 1). Cell viability was maintained over 70% in all conditions within the first 23 h, in compliance with predetermined specifications (Table 2). Thereon viability rates dropped in all conditions, nonetheless, Plasmalyte 148 was the solution gaving superior results followed by Ringer lactate and glucoside solution (74.7%, 68.6%, and 53.3%, respectively) (Fig. 1a, b). Cellular phenotype was determined daily without observing an alteration of MSC identity or differences between groups along time in any of the tested conditions (Table 3). Given these results, Plasmalyte 148 was selected as the electrolyte solution for subsequent experiments.

**Table 1:** Summary of conditions tested in the assessment of the effect of electrolyte solutions and albumins on MSC

MSC product	Electrolyte solution	Albumin type (w/v)
Fresh		
	Ringer lactate	2% HSA
	Glucoside	2% HSA
Cryopreserved	Plasmalyte 148	2% and 5% HSA
	Plasmalyte 148	2% and 5% AlbIX
	Plasmalyte 148	2% and 5% Recombumin Alpha

Different formulations were generated considering potential effects on fresh and cryopreserved products

**Table 2:** Specifications for the release of clinical grade BM-MSC for clinical use

Critical quality attribute	Acceptance criteria
Viability	≥ 70%
Identity	≥ 95% CD105, CD73, CD90 ≤ 5% CD45 and CD31 ≤ 20% *HLA-DR
Potency	Osteogenic, chondrogenic and adipogenic potential in vitro

Specifications in accordance to Phase I/II clinical trial (EudraCT No. 2010-024041-78). *HLA-DR levels only as informative parameter

**Table 3:** Phenotypic analyses of MSC resuspended in three different electrolyte solutions in the presence of 2% (w/v) HSA

Time	Day 0				Day 1				Day 2			
	CD45^−^/CD105^+^	CD31^−^/CD73^+^	HLA-DR^−^	CD90^+^	CD45^−^/CD105^+^	CD31^−^/CD73^+^	HLA-DR^+^	CD90^+^	CD45^−^/CD105^+^	CD31-/CD73^+^	HLA-DR^−^	CD90^+^
Plasmalyte 144	99.9	99.9	81.7	99.2	99.9	99.9	81.36	99.33	98.83	99.31	89.53	99.56
Ringer	99.9	99.9	73.8	99.4	99.9	99.8	78	98.99	99.86	99.63	86.23	99.73
Glucoside	99.8	99.8	83.5	98.7	99.8	99.8	80.1	98.68	99.82	99.79	87.51	99.54

Expression of markers is presented as a percentage of the ratio of cells positive/negative for each one

HSA, human serum albumin; MSC, multipotent mesenchymal stromal cell

### Effect of albumins on stability of fresh MSC

In the next series of experiments, the effect of recombinant (AlbIX and Recombumin Alpha) and serum-derived (HSA) albumin supplementation on MSC’s stability was assessed in a stability study performed at 2–8 °C with freshly prepared cellular suspensions. Viability and cell recovery were determined in samples taken from syringes containing 6.5 × 10^6^ MSC/mL in a final volume of 2.8 mL: 2 mL cell suspension of Plasmalyte 148 supplemented with 2% (w/v) albumin, and additional 0.8 mL of air volume (to facilitate homogenisation at each time point due to the sedimentation of cells in high concentrated suspensions). All conditions showed similar high viability rates within the first 44 h (98.5 ± 0.5%, 99.1 ± 0.3% and 99.1 ± 0.2%, for AlbIX, Recombumin Alpha, and HSA, respectively) and no significant differences observed.

### Effect of albumins on MSC post-thawing stability

**Fig. 2:**
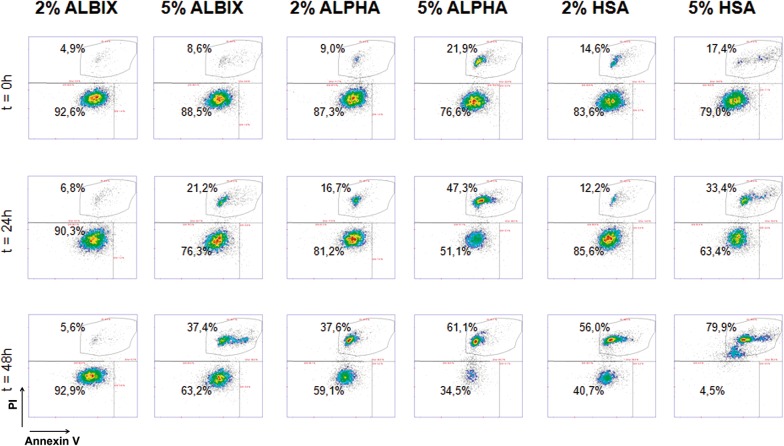
Effect of albumin type and concentration on the viability of cryopreserved MSC. Cryopreserved MSC were thawed using either one of the albumins at the concentrations (w/v) stated in the image. One cell line was used for all conditions tested. HSA, human serum albumin; MSC, multipotent mesenchymal stromal cells; PI, propidium iodide

Since freeze/thaw cycles commonly lead to low cells recovery, we studied next whether supplementation with yeast-derived recombinant albumins performed better than HSA on the stability of cells subjected to cryopreservation. For this purpose, MSC cultures were first scaled up and cryopreserved with 10% DMSO in Plasmalyte 148 supplemented with 2% or 5% (w/v) of each one of the albumins under examination. Then cells were thawed using the same supplement and concentration employed in the cryosolution. At 0 h, all albumin samples gave similar results, however by 24 h AlbIX was showing an improvement in viability. This was even more evident at 48 h with AlbIX still showing a viability of 92.9%. Recombumin Alpha, the other recombinant albumin, had reduced viability to 59.1% while HSA had the lowest viability at 40.7%. Interestingly, the use of higher albumin concentration, at 5% (w/v) showed a detrimental effect on cellular viability, particularly at late time points (Fig. 2).

**Fig. 3:**
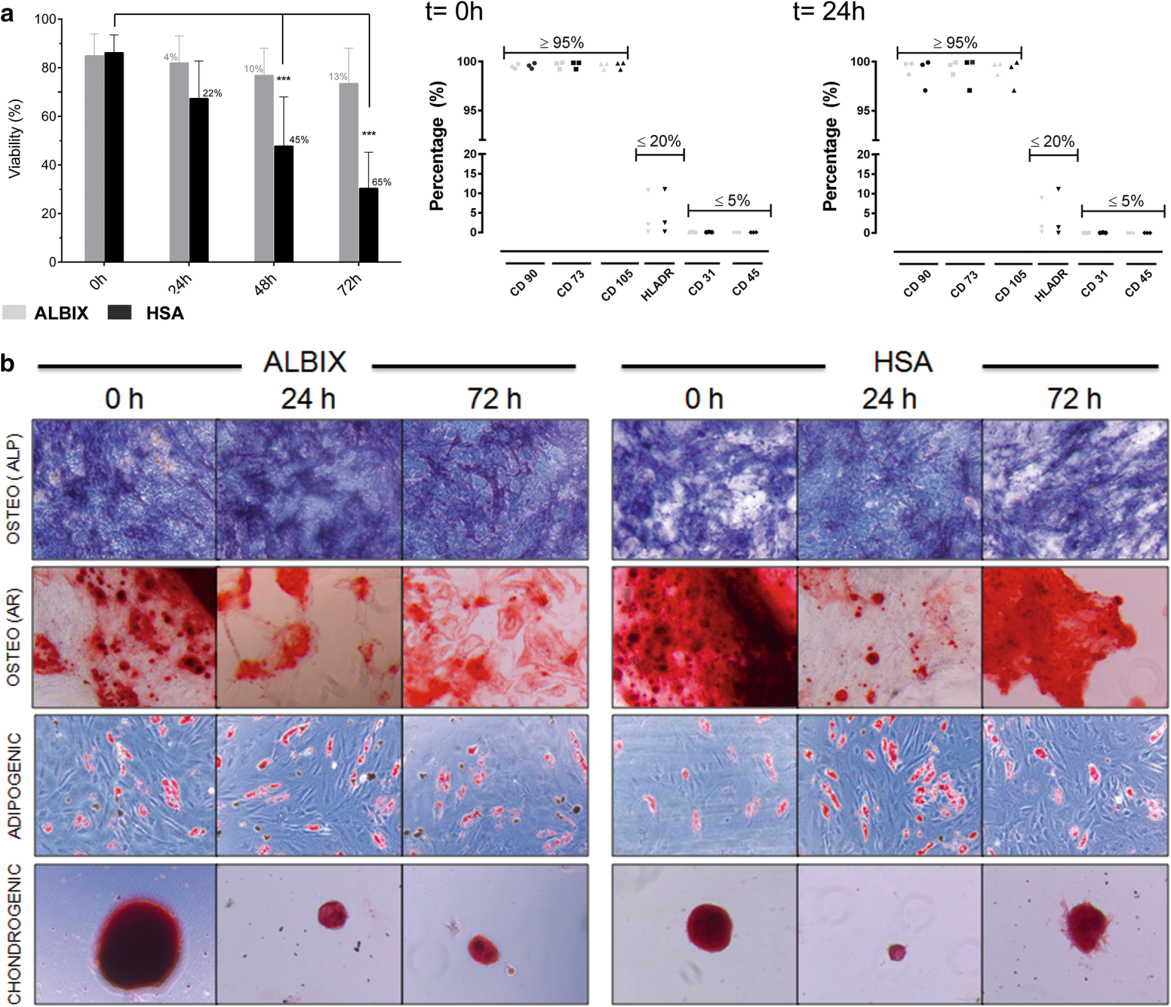
Retention of viability, phenotypic profile and multipotentiality of MSC after cryopreservation in the presence of either HSA or recombinant albumin. **a** Post-thawing stability assessed by flow cytometry showing the mean of the percentage of viability (%) along the stability follow up time using HSA (in black) or AlbIX (in grey) additives (n = 6 cell lines), percentage of reduction and results of ANOVA Tukey Multiple comparison test are showed; Phenotypic characterisation of MSC at times 0 and 72 h. Finally, in **b**, in vitro differentiation assays were performed to confirm osteogenic, adipogenic and chondrogenic potential of MSC and the outcome of each differentiation were assessed by specific stainings: alkaline phosphatase (ALP) and Alizarin Red (AR) for bone tissue, Oil Red O for fat; and Safranin O, for cartilage

In order to confirm these observations, we repeated the experiment using only 2% (w/v) AlbIX and HSA conditions in triplicates, analysed MSC identity and multipotency, and extended the follow up time period to 72 h. Remarkably, AlbIX contributed to preserve cell viability above 70% up to 72 h, which represents a significant increase of the product shelf-life as compared to standard conditions using HSA (Fig. 3a). Despite of the reduction of viability and total cell number over time, MSC maintained unaltered phenotype with time in both experimental conditions, as revealed by expression of CD73, CD90 and CD105, while lacking expression of CD31 and CD45 and having low level expression of HLA-DR (Fig. 3b, c). They also retained their multipotentiality and ability to differentiate into fat, cartilage and bone lineages (Fig. 3d and Additional file 1: Table S1).

### Effect of albumins on MSC under stressing conditions

**Fig. 4:**
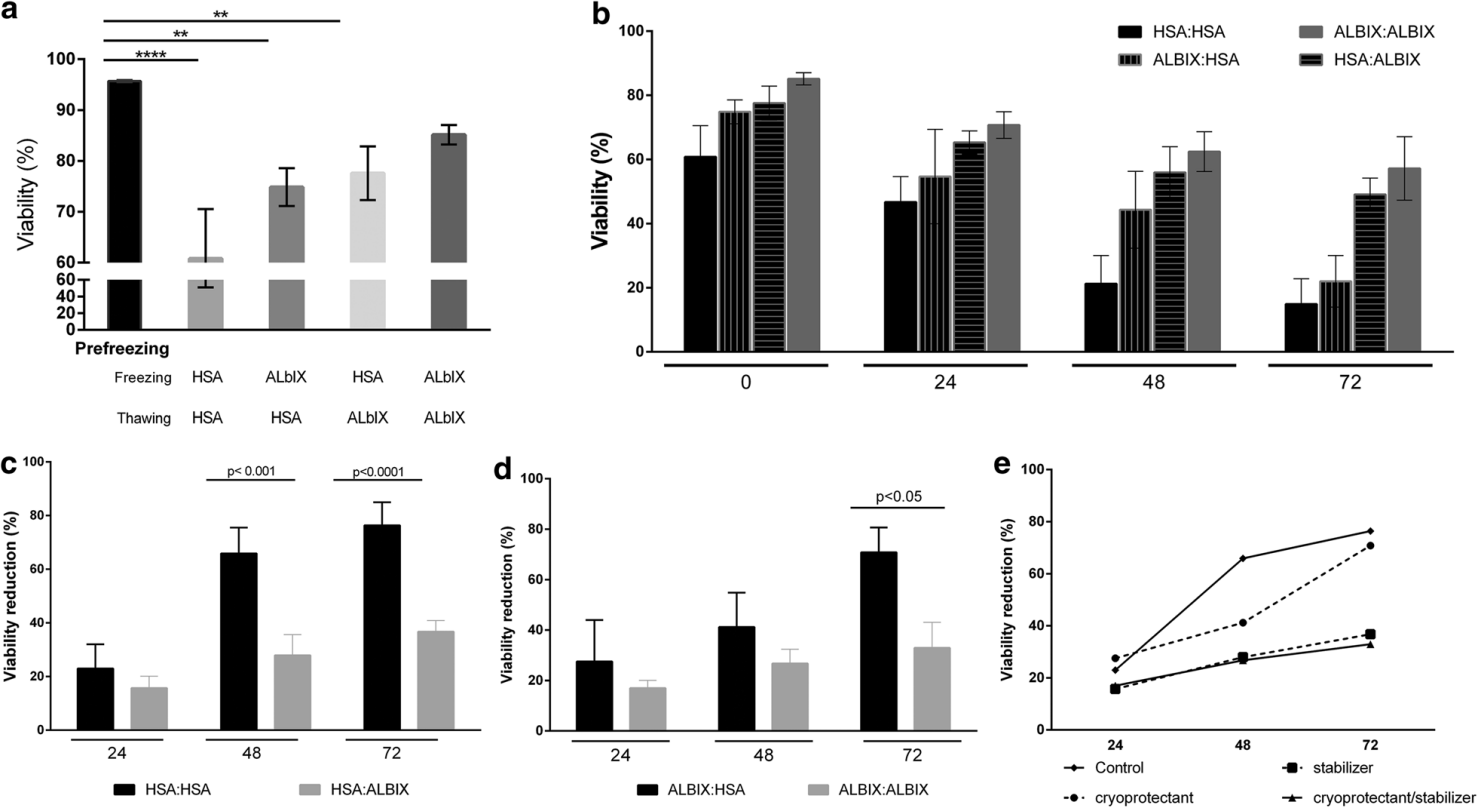
Impact of albumin supplementation when used as cryoprotectant, or additive, or both. Cryopreservation of MSC was performed using either AlbIX or HSA and followed by thawing and conditioning with either one of the two albumins generating the 4 possible combinations (HSA:HSA, HSA:AlbIX, AlbIX:AlbIX, and AlbIX:HSA). Viability pre- and post-cryopreservation shows higher viability rates in samples supplemented with AlbIX (**a**). Viability of all four conditions were followed up for 72 h (**b**). Data resulting from these experiments have been grouped according to the cryoprotectant used, either HSA (**c**) or AlbIX (**d**). In **e**, viability reduction is presented for all conditions along time, namely: control (HSA:HSA), as a cryoprotectant (AlbIX:HSA), as a stabilizer post-thawing (HSA:AlbIX), or both (AlbIX:AlbIX)

Next, we performed a series of experiments to interrogate whether the beneficial effect observed was restricted either to the cryopreservation stage or the after thawing period, or both. To do this, cells were cryopreserved in the presence of 2% (w/v) albumins (either AlbIX or HSA) and thawed using solutions supplemented with one of the albumins. Therefore, four different experimental conditions were generated, and all possible combinations with regards to supplementation of “cryosolution” and”thawing solution” were created (that is, HSA:HSA, AlbIX:HSA, HSA:AlbIX, AlbIX:AlbIX). The biggest drop in cell viability was observed immediately after thawing for the reference condition (HSA:HSA) and the most favourable conditions were those in which cells were both cryopreserved and thawed in solutions supplemented with AlbIX (Fig. 4a).

Immediately after thawing, all conditions tested (that is, HSA:HSA, AlbIX:HSA, HSA:AlbIX, AlbIX:AlbIX) supported viability values higher than 50%, thus falling within specifications (Table 2). However, significant differences were observed between the viability of cells before freezing and post-thawing (Fig. 4a). Interestingly, the more the cells were in contact with AlbIX, the higher the viability, with the AlbIX:AlbIX condition the one resulting in the highest viability post-thawing as compared to viability in HSA:HSA, AlbIX:HSA, and HSA:AlbIX conditions (85.2 ± 1.9% vs. 60.8 ± 9.7%, 74.9 ± 3.7%, and 77.6 ± 5.3%, respectively).

Thereon, viability was evaluated daily until day 3. Provided that different percentages of viability were observed at time point 0 h for each condition, data were normalized to initial values in order to evaluate the variation of viability over time thus facilitating cross comparison. Conditions were compared according to the type of albumin used in the cryopreservation solution. In this regard, HSA:AlbIX conditions showed higher percentage of viability all than HSA:HSA over time (Fig. 4b). Although no significant differences in viability between conditions cryopreserved with HSA were observed immediately after thawing, a significant difference in viability appeared from the 48 h time point (p < 0.001 at both t = 48 and 72 h). In the second set of conditions, using AlbIX as a supplement in the cryopreservation step, the use of AlbIX:AlbIX showed higher viability with time compared to AlbIX:HSA, a difference that became statistically significant at the 72 h time point (p < 0.05) (Fig. 4c).

Taking all the stability results together, it is evident that the more AlbIX is used in both stages of cryopreservation and thawing, the lowest reduction in viability with time is observed (Fig. 4d).

### Apoptosis studies

**Fig. 5:**
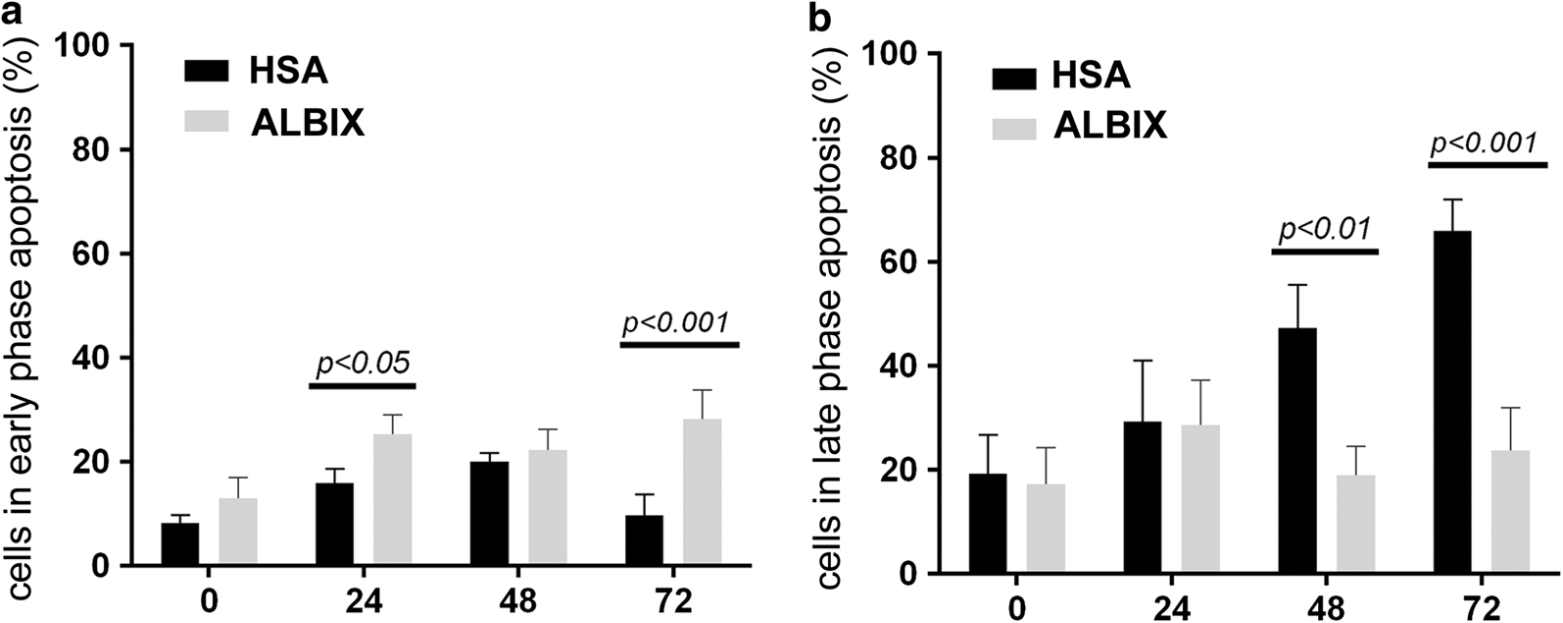
Analyses of apoptosis of cryopreserved MSC. Conditions tested were HSA:HSA and AlbIX:AlbIX and demonstrated that cryopreservation and post-thawing conditioning with Plasmalyte 148 supplemented with AlbIX extended the shelf-life of hMSC by preserving cells in the early apoptotic stage. Bars represent the percentage of early (**a**) and late (**b**) apoptotic cells for HSA and AlbIX cryopreservation conditions (n = 3 cell lines), ANOVA parametric Sidak’s multiple comparison tests were performed

In order to rule out potential mechanisms involved in the protection of cells by albumins, the apoptotic state was determined in HSA:HSA and AlbIX:AlbIX conditions.. Immediately after thawing, no significant differences were observed with regards to the percentage of early and late apoptotic cells between HSA:HSA and AlbIX:AlbIX. However, a significant difference was observed in early apoptotic cell number from the 24 h time point (Fig. 5a, b). Interestingly, the percentage of early apoptotic cells in HSA:HSA condition remained low whereas the AbIX:AlbIX condition showed a higher percentage that was clearly significant at the 72 h time point (p < 0.001). This observation correlated with an increase of the cell percentage in late apoptosis in the HSA:HSA condition as opposed to very low percentage of cells in the AlbIX:AlbIX condition, which was significantly lower from the 48 h time point (p < 0.001 at 48 h and p < 0.001 at 72 h) (Fig. 5b). In summary, the increased number of early apoptotic cells and reduced number of late apoptotic cells in AlbIX:AlbIX compared to HSA:HSA may suggest that supplementation with AlbIX slows down the progression of apoptosis, with less cells entering in late apoptosis.

## Discussion

Advances in the development of living cell-based therapies poses several challenges in the development of bioprocesses and product formulation due to the fragile nature of the drug substance [8]. Consequently, developers strive to understand and control all steps of the entire value chain design (from harvesting, upstream culture and cell preservation, to cell therapy administration) in order to define optimal conditions that enhance cell viability and reduce batch-to-batch variability, which are key for ensuring the quality of MSC-based products [9]. Cryopreservation is a crucial step for commercial stem cell therapies as it alleviates the need for a continued process thus allowing for flexibility [10]. Indeed, cryopreservation of MSC for banking off-the-shelf products and making them immediately available to patients is necessary in order to avoid a dependence on preparation time for each dose, allowing for quality control and product standardisation with multiple doses of the same, regulatory approved cell preparation. As an example of this, Prochymal is a cryopreserved cell-based medicine, whose drug substance is composed of ex vivo cultured adult human MSC suspension using 10% DMSO and 5% HSA in Plasma-Lyte A produced in large batches up to 10,000 doses expanded from a single donor [11]. Disappointing results of the Phase III study for Prochymal could possibly be related to freezing and thawing protocols, which could affect both MSC viability and functionality. Indeed Moll and collaborators demonstrated recently that critical quality attributes of MSC were altered upon freeze/thaw cycles [12], which was later supported by mechanistic studies showing that heat shock responses of MSC to cryopreservation were responsible of such observations [13].

Remarkably our experiments demonstrated that increasing the concentration of albumin from 2 to 5% (w/v) was not required for optimal cell stabilisation. In contrast, enhanced viability was observed when yeast-derived recombinant albumin was used instead of HSA. The advantage of albumin in cryopreservation is probably affected by purity and source, and this may explain why recombinant albumin prevented MSC from progressing into late stages of apoptosis upon thawing compared to using HSA. Since albumin is a carrier of many compounds present in the blood, these will be found in HSA from plasma but not in yeast-derived recombinant albumins. Therefore, slight changes in the structure and composition of albumins can affect their properties as excipients, leading to substantial changes in the interaction between cells and excipients and consequently affecting the stability of the final product.

From a regulatory perspective, switching the HSA initially proposed for a Phase I/II clinical trial to a recombinant albumin is a relevant modification that must be validated to demonstrate that each step in the manufacturing process results in a product falling within acceptable specifications on a consistent basis [6, 7, 14]. Our data show that using yeast-derived recombinant instead of plasma-derived albumin does not jeopardise the viability, identity and potency of the final product, and allows for a final product that contains only MSC without supplementation with any other human component.

## Conclusions

Critical quality attributes of MSC resuspended in a solution composed of 2% (w/v) AlbIX in Plasmalyte 148 electrolyte solution were maintained for at least 72 h post-thawing at 4–8 °C, thus improving its shelf-life as compared to formulations using HSA that dropped below 70% viability after 24 h. Moreover, the mode of action of AlbIX protection relates to the prevention of cells from progressing into late apoptosis.

## Methods

### Cells and cell culture

Clinical grade BM-MSC were produced within the context of a clinical trial (EudraCT No. 2010-024041-78) with appropriate donor informed consent for use in research [15]. Cells were further expanded in vitro up to sufficient numbers by using Dulbecco’s Modified Eagle’s Medium (DMEM, Gibco) supplemented with 10% human serum B (hSerB; Banc de Sang i Teixits) containing 2 mM glutamine in T-flasks and CellSTACKS (CellSTACKs (Corning Incorporated Life Sciences) at 1 × 10^3^ to 3.5 × 10^3^ cells/cm^2^ seeding density [16]. All cultures were maintained at 37 °C and 5% CO_2_ in humidified incubators.

### Experimental design

The effect of electrolyte solutions (5% glucoside solution, Grifols; Ringer lactate, Fresenius Kabi; and Viaflo Plasmalyte 148, Baxter) and albumins (recombinant albumins: AlbIX^®^ and Recombumin^®^ Alpha, from Albumedix; and HSA: Albutein, from Grifols) were used to evaluate their effect on the preservation of CQA in MSC, including cell recovery, viability, identity and potency, both pre- and post-thawing, to assess the stability of the final reconstituted cellular product (Table 1).

Cells were cryopreserved in a solution composed of Dulbecco’s Phosphate-Buffered Saline (DPBS; Gibco) supplemented with 10% (v/v) dimethyl sulfoxide (DMSO; OriGen Biomedical, Austin, TX, USA) and 2% (w/v) human serum albumin (HSA; Grifols, Barcelona, Spain), by applying a controlled freezing rate of 1 °C/min in a Mr. Frosty device (Nalgene, Rochester, NY, USA) kept in a − 80 °C freezer for 24 h before storage at − 196 °C in a liquid nitrogen tank until further use [17]. On the day of thawing, cells were rapidly thawed in a 37 °C water bath, then slowly diluted 1:10 using pre-cooled thawing solution consisting of 2% (w/v) albumin in Plasmalyte 148. DMSO was washout by centrifugation at 340*g* for 10 min. Finally, each experimental condition for assessing stability was created by resuspending in Plasmalyte 148 supplemented with 2% (w/v) of either one of the albumins and set up 10 in mL syringes.

### Differentiation assays

Specific StemPro differentiation media (Gibco) were used for the osteogenic, chondrogenic and adipogenic induction of undifferentiated MSC cultures in vitro. Safranin O (Sigma), Oil Red O (Sigma), Alkaline Phosphatase (Takara Bio Inc.), and Alizarin Red (Sigma) stainings were performed for the determination of the outcome of the differentiation assays [18, 19].

### Cell count, viability and apoptosis

Cells were counted either by following the Trypan blue dye exclusion methods or by using Perfect-Count Microspheres (Cytognos) in a FACSCalibur cytometer (Becton–Dickinson). Viability was determined using the 7-Amino-Actinomycin D (7-AAD, BD Biosciences) exclusion method and expressed as a percentage (%) of total cells. Data were analyzed with the CellQuest Pro (Becton–Dickinson) software. Occurrence of apoptosis and the apoptotic stage (either early or late apoptosis) was determined on a NC3000™ Nucleocounter (Chemometec, Copenhagen, Denmark) using a double staining procedure with Annexin V and propidium iodide (PI), following the manufacturer’s instructions. Early apoptosis stage is characterized by the translocation phosphatidylserine (PS) in the cell membrane, which was detected by Annexin V specific binding to PS. Later on in the apoptosis progression, membrane intergrity loss occurs which in this study was detected by the penetration of the impermanent dye PI additionaly to the Annexin V.

### Phenotype assessment

Immunophenotypic characterization of BM-MSC was performed using the following antibodies: mouse anti-human CD45-fluorescein isothiocyanate (CD45-FITC, HI30, BD Pharmingen), anti-human CD105-phycoerythrin (CD105-PE, 43A4E1, Miltenyi Biotec), anti-human HLA-DR-FITC (L243, BD Biosciences), anti-human CD90 PE (F15-42-1-5, Beckman Coulter), mouse anti-human CD31-FITC (WM59, BD Pharmingen) and mouse anti-human CD73 PE (AD2, BD Pharmingen). Cells were stained for 15 min at room temperature, washed and resuspended in phosphate-buffered saline (PBS; Invitrogen). Non-specific cell staining was ruled out by using mouse immunoglobulin isotype controls (BD Pharmingen). Acquisition was done using a FACSCalibur and data were analyzed with the CellQuest Pro software.

### Data analysis

Descriptive data were expressed as mean ± standard deviation. ANOVA multiple comparison tests were used to determine differences between experimental conditions taking into account all parameters. Statistical significance was set at: *p < 0.05; **p < 0.01; ***p < 0.001; and ****p < 0.0001.

## Additional file

**Additional file 1: Table S1.** Differentiation potential of MSC. The potential to differentiate into the chondrogenic, adipogenic and osteogenic lineages is maintained with the use of both AlbIX and HSA supplements after a freeze/thaw cycle. The symbols represent the graduation of the staining as: − = no differentiation; + = low, ++ = medium, and +++ = high. NP = Not performed; ALP = Alkaline Phosphatase; AR = Alizarin Red).

## References

[1] Fischbach MA, Bluestone JA, Lim WA (2013). Cell-based therapeutics: the next pillar of medicine. Sci Transl Med..

[2] Galvez-Martin P, Hmadcha A, Soria B, Calpena-Campmany AC, Clares-Naveros B (2014). Study of the stability of packaging and storage conditions of human mesenchymal stem cell for intra-arterial clinical application in patient with critical limb ischemia. Eur J Pharm Biopharm.

[3] Dimarino AM, Caplan AI, Bonfield TL (2013). Mesenchymal stem cells in tissue repair. Front Immunol..

[4] Sensebe L, Gadelorge M, Fleury-Cappellesso S (2013). Production of mesenchymal stromal/stem cells according to good manufacturing practices: a review. Stem Cell Res Ther..

[5] Fanali G, di Masi A, Trezza V, Marino M, Fasano M, Ascenzi P (2012). Human serum albumin: from bench to bedside. Mol Aspects Med.

[6] Committee for medicinal products for human use (CHMP) European Medicines Agency, EMEA/CHMP/QWP/396951/2006; 2007. p 1–12.

[7] Guideline on excipients in the dossier for application for marketing authorisation of a medicinal product EMEA/CHMP/QWP/396951/2006 (2007).

[8] Schneider CK, Salmikangas P, Jilma B, Flamion B, Todorova LR (2010). Challenges with advanced therapy medicinal products and how to meet them. Nat Rev Drug Discov..

[9] Erben RG, Silva-Lima B, Reischl I, Steinhoff G, Tiedemann G, Dalemans W (2014). White paper on how to go forward with cell-based advanced therapies in Europe. Tissue Eng Part A..

[10] Hunt CJ (2011). Cryopreservation of human stem cells for clinical application: a review. Transfus Med Hemother..

[11] Galipeau J (2013). The mesenchymal stromal cells dilemma–does a negative phase III trial of random donor mesenchymal stromal cells in steroid-resistant graft-versus-host disease represent a death knell or a bump in the road?. Cytotherapy..

[12] Moll G, Alm JJ, Davies LC, Von Bahr L, Heldring N, Stenbeck-Funke L (2014). Do cryopreserved mesenchymal stromal cells display impaired immunomodulatory and therapeutic properties?. Stem Cells..

[13] Francois M, Copland IB, Yuan S, Romieu-Mourez R, Waller EK, Galipeau J (2012). Cryopreserved mesenchymal stromal cells display impaired immunosuppressive properties as a result of heat-shock response and impaired interferon-gamma licensing. Cytotherapy..

[14] Martin PG, Martinez AR, Lara VG, Naveros BC (2014). Regulatory considerations in production of a cell therapy medicinal product in Europe to clinical research. Clin Exp Med..

[15] Soler R, Orozco L, Munar A, Huguet M, Lopez R, Vives J (2016). Final results of a phase I–II trial using ex vivo expanded autologous mesenchymal stromal cells for the treatment of osteoarthritis of the knee confirming safety and suggesting cartilage regeneration. Knee..

[16] Codinach M, Blanco M, Ortega I, Lloret M, Reales L, Coca MI (2016). Design and validation of a consistent and reproducible manufacture process for the production of clinical-grade bone marrow-derived multipotent mesenchymal stromal cells. Cytotherapy..

[17] Oliver-Vila I, Coca MI, Grau-Vorster M, Pujals-Fonts N, Caminal M, Casamayor-Genesca A (2016). Evaluation of a cell-banking strategy for the production of clinical grade mesenchymal stromal cells from Wharton’s jelly. Cytotherapy..

[18] Caminal M, Velez R, Rabanal RM, Vivas D, Batlle-Morera L, Aguirre M (2017). A reproducible method for the isolation and expansion of ovine mesenchymal stromal cells from bone marrow for use in regenerative medicine preclinical studies. J Tissue Eng Regen Med..

[19] Vivas D, Caminal M, Oliver-Vila I, Vives J (2018). Derivation of multipotent mesenchymal stromal cells from Ovine Bone marrow. Curr Protoc Stem Cell Biol..

