# Supplementary material for: Stability enhancement of clinical grade multipotent mesenchymal stromal cell-based products
Source: J Transl Med. 2018 Oct 24;16:291. doi: 10.1186/s12967-018-1659-4 (PMC6201632; doi:10.1186/s12967-018-1659-4)
Supplement: Supplementary file 1 — Additional file 1: Table S1. Differentiation potential of MSC. The potential to differentiate into the chondrogenic, adipogenic and osteogenic lineages is maintained with the use of both AlbIX and HSA supplements after a freeze/thaw cycle. The symbols represent the graduation of the staining as: − = no differentiation; + = low, ++ = medium, and +++ = high. NP = Not performed; ALP = Alkaline Phosphatase; AR = Alizarin Red). [file 12967_2018_1659_MOESM1_ESM.docx]

**Additional Table S1. Differentiation potential of MSC**. The potential to differentiate into the chondrogenic, adipogenic and osteogenic lineages is maintained with the use of both AlbIX and HSA supplements after a freeze/thaw cycle. The symbols represent the graduation of the staining as: - = no differentiation; + = low, ++ = medium, and +++ = high. NP = Not performed; ALP = Alkaline Phosphatase; AR = Alizarin Red)

| MSC line | Condition | Time (h) | chondrogenic | adipogenic | osteogenic | |
| --- | --- | --- | --- | --- | --- | --- |
|  |  |  |  |  | ALP | AR |
| 1 | Albutein | 0 | + | + | +++ | + |
|  |  | 24 | + | ++ | +++ | + |
|  |  | 72 | + | ++ | +++ | +++ |
|  | AlbIX | 0 | ++ | ++ | +++ | + |
|  |  | 24 | ++ | ++ | +++ | ++ |
|  |  | 72 | + | ++ | +++ | +++ |
| 2 | Albutein | 0 | + | + | +++ | +++ |
|  |  | 24 | ++ | ++ | +++ | - |
|  |  | 72 | NP | ++ | ++ | +++ |
|  | AlbIX | 0 | + | ++ | +++ | +++ |
|  |  | 24 | ++ | ++ | +++ | +++ |
|  |  | 72 | + | ++ | ++ | +++ |
| 3 | Albutein | 0 | ++ | - | +++ | + |
|  |  | 24 | ++ | - | +++ | - |
|  |  | 72 | NP | - | +++ | ++ |
|  | AlbIX | 0 | ++ | - | +++ | + |
|  |  | 24 | + | - | ++ | - |
|  |  | 72 | + | - | ++ | - |
